# Multi-objective hybrid optimized coil design for enhanced efficiency, improved voltage gain, and compactness for inductive power transfer

**DOI:** 10.1038/s41598-025-12741-w

**Published:** 2025-09-01

**Authors:** Kripalakshmi Thiagarajan, T. Deepa, Prabhakar Mahalingam

**Affiliations:** 1 School of Electrical Engineering, Vellore Institute of Technology, Chennai, India; 2 Centre for Smart Grid Technologies, Vellore Institute of Technology, Chennai, India

**Keywords:** Automated guided vehicles, Hybrid optimization, Inductive charging, Light electric vehicles (LEVs), And optimized coil design, Engineering, Electrical and electronic engineering

## Abstract

With a rising global population and vehicle usage, electric vehicles (EVs) have emerged as a sustainable solution for carbon neutrality. The paper’s main objective is to test the performance of the optimized coil design for the Inductive power transfer (IPT) prototype designed for 48 V light EV (LEV) applications operating at 86 kHz. The coils are optimized for objectives such as compactness and power transfer efficiency, using a hybrid multi-objective optimization algorithm combining Taylor-series tuning and Dove Swarm (DSO) optimization. The optimized coil design achieved power transfer efficiency (PTE) of 94% with a voltage gain of 0.93 and current gain of 0.9. A minimum drop of approximately 0.7 V (1.5%) is observed between the primary to secondary coils. Simulation and hardware tests showed minimal voltage loss and strong coupling, validated for variable frequency operation. This shows the robustness of the LC optimized coil compact IPT system designed in this paper. Compact coil design in this research aids in high power transfer efficiency while reducing size, making it well-suited for LEV wireless charging.

## Introduction

In the realm of wireless power transfer (WPT), a notable advantage lies in the ability to charge devices without any physical contact. The incorporation of WPT technologies presents a viable solution to mitigate various risks and drawbacks associated with plug-in charging. WPT is classified into two fundamental categories: radiative and non-radiative, based on the principles of electromagnetic waves and electromagnetic coupling, respectively. The radiative category is termed “far-field,” while the non-radiative category is termed “near-field”^1–3^. The near-field is based on the principle of mutual inductance (M) to establish coupling between the transmitter and receiver coil separated by an air gap by creating an electric, magnetic, or electromagnetic field to aid in power transfer.

While author Luke Hutchinson et al.^4^ stated that inductive coupling is limited to mere inches, other researchers, Giulia Di Capua et al.^5^, Chirag Panchal et al.^6^, and Zicheng Bi et al.^7^ investigated the transmitter (T_X_) and receiver (R_X_) coils along with power electronic interfaces (PEIs) that can improve the overall performance of the IPT system.

In the configuration of the IPT charging circuit, the primary side consists of a high-frequency (HF) inverter, a compensation circuit, and a primary coil (T_X_), and the secondary side consists of a secondary coil (Rx), rectifier, and battery load. The high-frequency AC for IPT uses an inverter, and selecting the topology needs a detailed review. Voltage source inverter (VSI) seems to be promising for IPT applications in maintaining a stable output when the load changes, and as Mai, R^8^ and A. T. Al-Awami et al.^9^ studies prove that the VSI is relatively insensitive to changes and makes it suitable for ideal dynamic charging conditions. Z. Liao^10^ has identified Class-E resonant inverters as suitable for IPT systems operating at higher frequencies in the MHz range^10,11^. A new topology idea for dual-frequency operation of a resonant inverter is presented in^12^. Voltage stress is notably high, approximately 3.5 times greater than the input voltage, as demonstrated by C. Carretero^13^.

Capacitive compensation is necessary to reduce the power supply’s VA rating and mitigate leakage current issues arising from the misalignment of loosely connected coils. Extensive research in IPT applications has led to the investigation, analysis, and development of various compensation networks for addressing leakage inductance problems^14,15^. By aligning the coils with added LC circuit frequency and the switching frequency of the inverter, the frequency splitting issue is resolved.

The performance of four capacitor compensation topologies in a loosely connected inductive power transfer system with a Class E amplifier, as presented in^16^, is examined in this research. Inductive power transfer frequently uses a capacitor compensation approach to address the issue of significant leakage inductance and to achieve maximum power transfer. The properties of the LC, LCL, and LCLC topologies are realized through comparative studies in the literature. Hybrid compensation networks, such as LC-CLC, enhance coupler misalignment performance^17^. A high-power IPT system with higher-order compensation on the primary side demonstrates high efficiency, as seen in^15^ by LCL-L and in^18^ by LCC-S compensation networks. Author Yang et al.^19^ showed the effectiveness of LC compensation at variable frequency operation.

The helical coil has better coupling compared to the spiral coil when the air gap widens^20,21^ and using the combined coil structure results in improved performance. A comparison of integrated coils with ferrite bars has proven to be a promising magnetic coil geometry for the IPT system^22,23^. Coils designed with U cores, E cores, and pot cores were initially investigated, however, they were unsuitable for EV applications because of their thickness.

In this article, the layout is structured into three primary sections, the second section delves into the examination of equations concerning frequency characteristics, voltage, and power rating, and design equations. These equations serve as the foundation for establishing IPT parameters for simulation models and subsequently for experimental prototype modeling.

The third section explores the simulation result with visualization of the voltage waveforms at various stages of the IPT system, designed with a power rating of 288 W, with a frequency range chosen to be 86 kHz. It also provides an overview of the optimization approach proposed in the article and the performance comparison of the proposed optimization algorithm with other algorithms in the literature.

The final section presents the performance aspects of the HF inverter, analyzing switching waveforms, rise and fall times, and device voltage characteristics. Furthermore, we scrutinize the experimental results with the inverter voltage response and explore AC voltage transfer between primary and secondary coils under conditions of LC compensation with variations in frequency. Experimental results in this section serve to validate our findings and discussions.

## Formulation of design equations

**Fig. 1:**
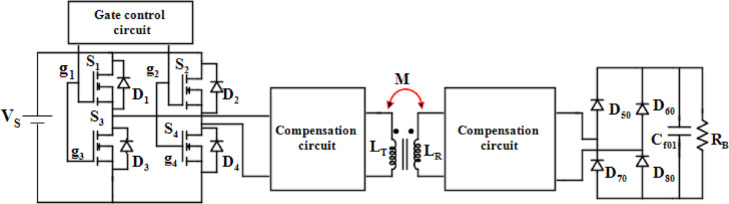
Circuit configuration of IPT.

The general configuration of IPT consists of several components for various power conversion stages for AC and DC power transfer and regulation. The circuit is depicted in Fig. 1, comprising an HF inverter (S_1_ to S_4_), an AC to DC rectifier stage (D_10_ to D_80_), compensation circuits, and a loosely coupled transformer (L_T_, L_R_). The compensation circuits range from two capacitor combinations to T-type networks with LC combinations.

**Fig. 2:**
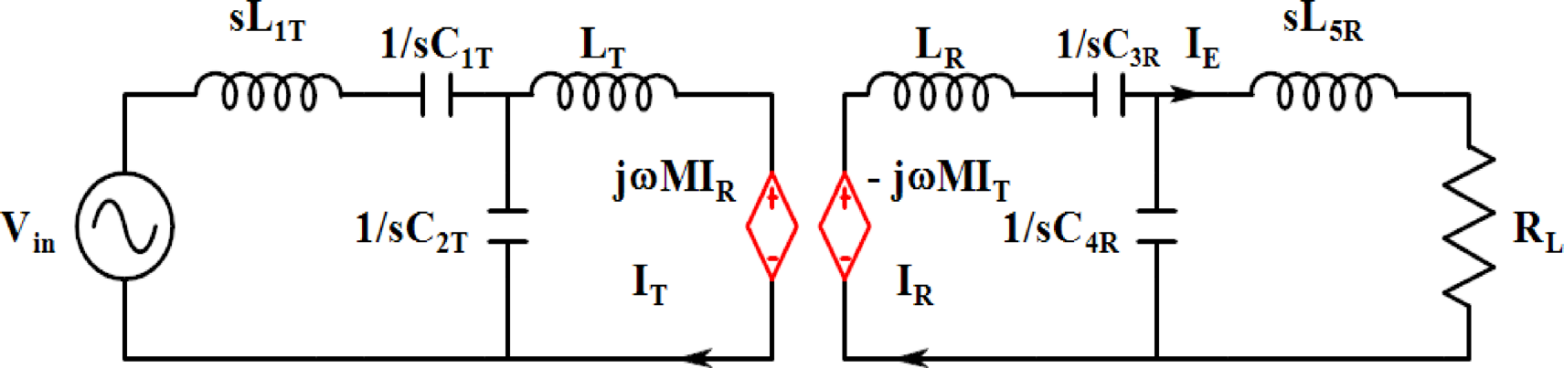
Equivalent circuit for T-type network analysis.

Although the use of double-sided compensation topologies is a recent trend, single-sided primary tuning^24,25^ exhibits a high voltage transfer ratio, as demonstrated in the article with experimental results. Fig. 2 illustrates the equivalent circuit with an LC-type compensation network for voltage equations and for estimating resonating element parameters.

1 $$\begin{gathered} C_{{1T}} = \frac{{C_{{2T}} }}{{n^{2} }}~,~\left( {L_{T} - M} \right) = n^{2} \left( {L_{T} - M} \right),L_{T} = L_{R} = \left( {L_{T} - M} \right), \hfill \\ Z_{T} + Z_{M} = ~\frac{1}{{sC_{{1T}} }} + s\left( {L_{T} - M} \right) + sM~ = ~\frac{{s^{2} C_{{1T}} L_{T} + 1}}{{SC_{{1T}} }} \hfill \\ \end{gathered}$$

2 $$\:{\omega\:}_{01}=\frac{1}{\sqrt{{C}_{1\:T}{L}_{T}}}$$

3 $$\begin{aligned} Z_{{eq}} & = \left( {Z_{T} + Z_{M} } \right)\left| {\left| {\left( {Z_{R} } \right) = \frac{1}{{sC_{1} T}} + s\left( {L_{T} - M} \right) + sM~} \right|} \right|\frac{1}{{sC_{1} }} + s\left( {L_{T} - M} \right) \\ ~ & = \frac{{s^{2} C_{{1T~}} \left( {L_{T} - M} \right) + s^{4} C^{2} _{{1T}} L_{T} \left( {L_{T} - M} \right)C^{2} _{{1T}} }}{{1 + s^{2} C_{{1T~}} \left( {L_{T} - M} \right) + s^{2} C_{{1T~}} M + s^{2} C_{{1T~}} \left( {L_{T} - M} \right) + \frac{1}{{SC_{{1T}} }}}} \\ \end{aligned}$$

4 $$\:{\omega\:}_{02}=\frac{1}{\sqrt{{C}_{1\:}({L}_{T}-M)}}$$

5 $$\:{\omega\:}_{03}\:=\frac{1}{\sqrt{{C}_{1\:}({L}_{T}+M)}}\:\:$$

$\:{C}_{1T}\:$– Primary side capacitor​, scaled by the square of the turns ratio $\:n$

$\:{C}_{2T}-$ Secondary-side capacitor

$\:{L}_{T}-$ Self-inductance of the transmitter coil.

*M*– Mutual inductance between coils

$\:{L}_{R}-\:$Self-inductance of the receiver coil

$\:{Z}_{T}-$ Transmitter-side impedance

$\:{Z}_{M}-$ Mutual inductance-related impedance

$\:{Z}_{R}-$ Receiver-side reflected impedance

$\:{\omega\:}_{01}-$ Fundamental resonant frequency of the primary side

$\:{\omega\:}_{02}-$ Lower resonant frequency

$\:{\omega\:}_{03}-$ Higher resonant frequency

The analysis of the two-capacitor network is given in Eqs. (2), (3), (4), (5), while for the T-type network, the estimation of resonant elements is evaluated through Eqs. (6)–(14). The estimated capacitor value for a particular frequency operation can be calculated from Eqs. (2), (3), (4), (5), and using the expression Q = ωL/R with a specified quality factor (Q) value. This choice is based on the concept that high Q values correspond to a narrow frequency range of operation, while low Q values correspond to a broader frequency range.

6 $$\:{Z}_{T}+{Z}_{M}=\frac{{sL}_{1T}\times\:\frac{1}{{sC}_{2T}}}{{sL}_{1T}+\frac{1}{{sC}_{2T}}}+s\left({L}_{T}-M\right)+\frac{1}{s{C}_{1T}}+sM=\frac{{{s}^{2}L}_{1T}{C}_{1T}+{(s}^{2}{C}_{1T}{L}_{T})\left({s}^{2}{L}_{1T}{C}_{2T}+1\right)\:}{s{C}_{1T}\left({s}^{2}{L}_{1T}{C}_{2T}+1\right)}$$

7 $$\:{\omega\:}_{lcc01}=\frac{1}{\sqrt{\left.{C}_{1T}{L}_{1T}{\left(L\right.}_{T}{C}_{2T}\right)}}$$

8 $$\:{\omega\:}_{lcc02}=\frac{1}{\sqrt{{C}_{1}{L}_{P}\:}}$$

9 $$\:{\omega\:}_{lcc03}=\frac{1}{\sqrt{{C}_{P}{L}_{f}}}\:\:$$

10 $$\begin{gathered} ~\left. {Z_{{eq}} = \frac{{sL_{{1T}} \times \frac{1}{{sC_{{2T}} }}}}{{sL_{{1T}} + \frac{1}{{SC_{{2T}} }}}} + s\left( {L_{T} - M} \right) + \frac{1}{{sC_{{1T}} }} + sM~} \right\|\frac{{sL_{{1T}} \times \frac{1}{{sC_{{2T}} }}}}{{sL_{{1T}} + \frac{1}{{sC_{{2T}} }}}} + s\left( {L_{T} - M} \right) + \frac{1}{{sC_{{1T}} }} \hfill \\ = \frac{{\left[ {s^{2} L_{{1T}} C_{{2T}} + 1} \right]\left[ {s^{2} L_{{1T}} C_{{1T}} \left( {L_{T} - M} \right)} \right]\left[ {s^{2} L_{{1T}} C_{{1T}} \left( {L_{T} + M} \right)} \right]}}{{\left[ {s_{{C_{1} }} \left( {s^{2} L_{{1T}} C_{{2T}} + 1} \right)} \right]\left[ {\left( {s^{2} L_{{1T}} C_{{1T}} + 1} \right)} \right]}} \hfill \\ \end{gathered}$$

11 $$\:{\omega\:}_{lcc04}\:=\frac{1}{\sqrt{{C}_{2T}{L}_{1T}}}$$

12 $$\:{\omega\:}_{lcc05}\:=\frac{1}{\sqrt{{L}_{1T}{C}_{1T}\left({L}_{T}-M\right)}}$$

13 $$\:{\omega\:}_{lcc06}\:=\frac{1}{\sqrt{{L}_{1T}{C}_{1T}\left({L}_{T}+M\right)}}$$

14 $$\:{\omega\:}_{lcc07}\:=\frac{1}{\sqrt{{L}_{1T}{C}_{1T}}}$$

$\:s=j\omega\:-$ for Laplace transformation

$\:{C}_{3R},{C}_{4R}-\:$ Compensation capacitors on the receiver side

$\:{L}_{3R},{L}_{4R}-$Self-inductances of receiver side coils

$\:{\omega\:}_{lcc01}-$ fundamental resonant frequency

$\:{\omega\:}_{lcc02}-$ dominant frequency based on load-side resonance

$\:{\omega\:}_{lcc03}-$ secondary resonant frequency by filter components

$\:{\omega\:}_{lcc04}-$ resonant frequency under differential-mode coupling

$\:{\omega\:}_{lcc05}\:-$ resonant frequency under differential-mode coupling

$\:{\omega\:}_{lcc06}-$ resonant frequency under common-mode coupling

$\:{\omega\:}_{lcc07}-$ uncoupled resonance at the transmitter side

In the equivalent circuit shown in Fig. 2, the compensation capacitors and inductors are labelled as $\:{C}_{3R},{C}_{4R}$ and $\:{L}_{3R},{L}_{4R}$, for mathematical expression, the notation is taken as the same as the primary side with $\:{C}_{1T}$ and $\:{C}_{2T\:}$and $\:{L}_{T}$ and $\:{L}_{1T}$ respectively for resonant frequency derivations of the two types of compensation networks by using two-port network theory^26^.

**Fig. 3:**
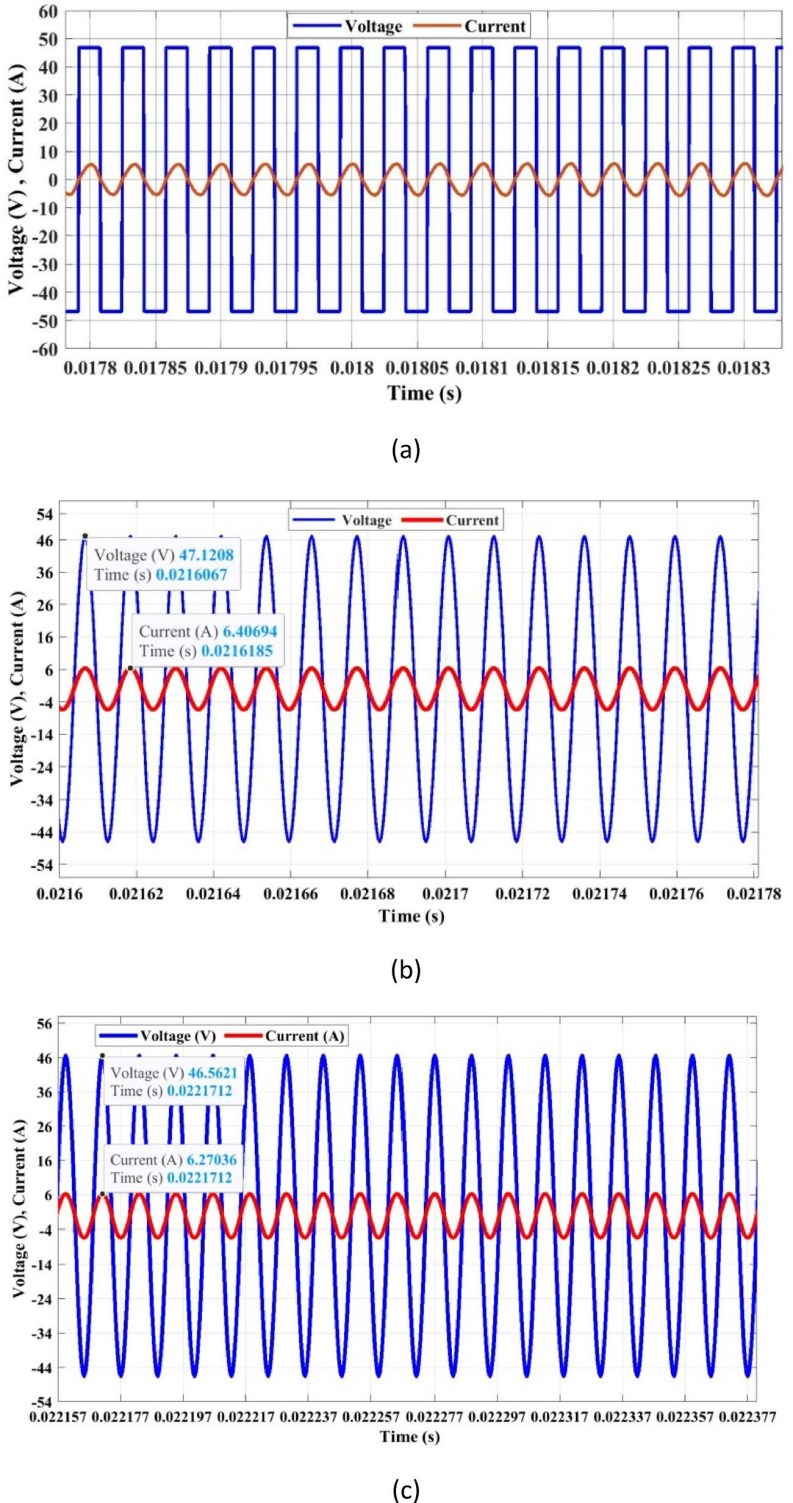

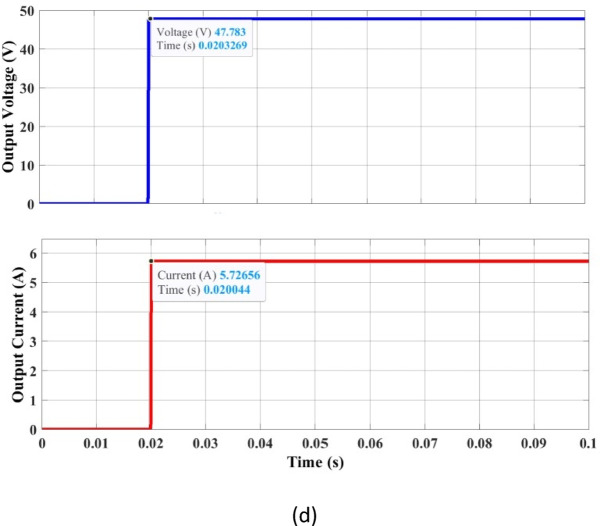
(**a**) Input voltage and current response (Inverter stage), (**b**) Transmitter side Voltage and Current, (**c**) Receiver side voltage and current, (**d**) Output DC regulated voltage and current.

By applying Kirchhoff’s laws to Fig. 3, the voltage equations are obtained, and Eqs. (15)–(19) provide the estimation of output current and resonant elements.

15 $$\:{V}_{in}=\left(j\omega\:{L}_{1T}-\frac{1}{j\omega\:{C}_{2T}}\right){I}_{in}-1/\left(j\omega\:{C}_{2T}\right){I}_{T}$$

On the secondary side, the voltage equation is expressed as,

16 $$\:j\omega\:M{I}_{T}=j\left(\omega\:{L}_{R}-\frac{1}{\omega\:{C}_{2T}}-\frac{1}{\omega\:{C}_{2S}}\right){I}_{R}\:+\:(j\frac{1}{\omega\:{C}_{4R}}{)I}_{E}$$

17 $$\:{I}_{E}=\:\frac{{\omega\:}^{3}{C}_{2T}{C}_{4R}M{V}_{in}}{j}=\:\frac{M{V}_{in}}{j\omega\:{L}_{1T}{L}_{5R}}$$

The resonant tank must be precisely designed to ensure that the phase of the input impedance attains absolute zero. Calculate the L and C elements of the compensation circuit from Eqs. (17), (18), and (19).

18 $$\:\omega\:{L}_{1T}-\frac{1}{\omega\:{C}_{2T}}=0\:$$

19 $$\left[ {j\left( {\omega L_{{1R}} - \frac{1}{{\omega C_{{2R}} }} - \frac{1}{{\omega C_{{4R}} }}} \right)} \right] = 0$$

$\:{V}_{in}-$ Input AC voltage to the transmitter

$\:{L}_{1T},\:{L}_{T}-$ Self-inductance of primary coils

$\:{C}_{1T},{C}_{2T}-$ Compensation capacitors on transmitter side

$\:{I}_{T},\:{I}_{R},\:{I}_{E}-$ Currents in transmitter, receiver, and load side, respectively

$\:{L}_{P},\:{C}_{P}-$ Equivalent inductance/capacitance used for lumped circuit analysis

**Table 1:** Calculated parameters and values.

Parameter	Value
Frequency (*f*)	86 kHz
Angular frequency ($\:\omega\:$)	540,000 rad/s
Inductance ( $\:{L}_{1T}$ $\:)$	110 µH
Resistance ($\:R$)	8Ω
Mutual inductance ($\:M$)	22 µH
Compensation capacitance $\:\left({C}_{1T}\right){C}_{1T}=\frac{1}{{\omega\:}^{2}{L}_{1T}}$	31.3 nF
Coupling coefficient ($\:k)$	0.2
Input voltage	48 V
Input current	6 A

## Validation of the system: simulation results

### Simulation response of the IPT system

**Fig. 4:**
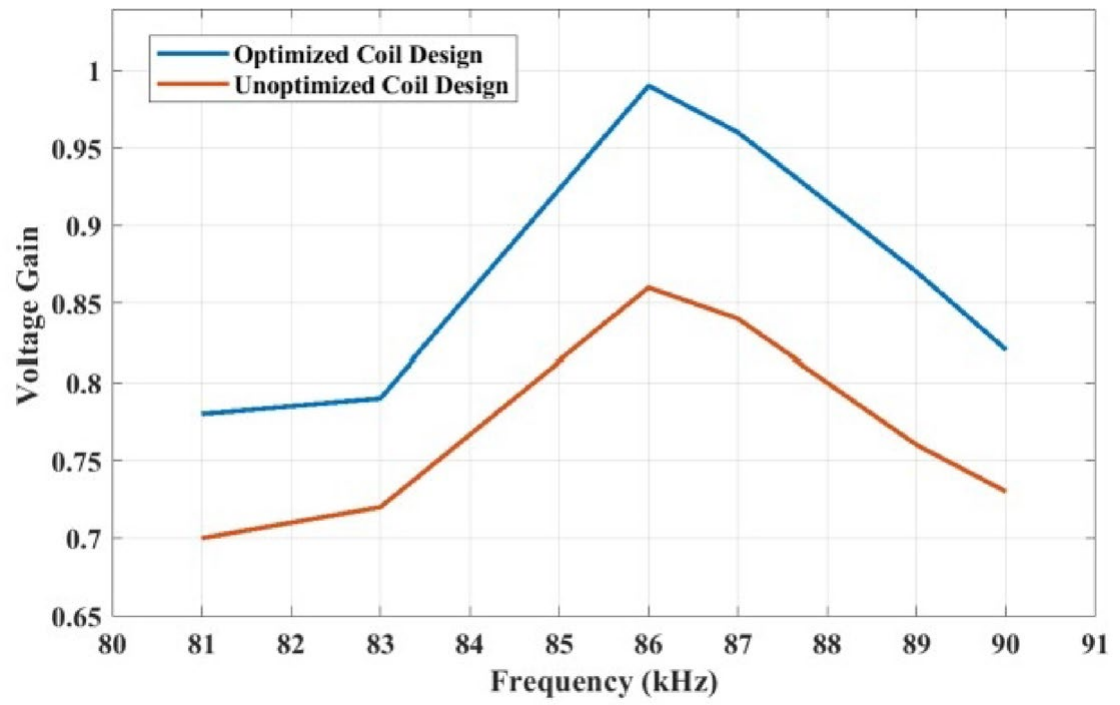
Optimized vs unoptimized gain vs frequency response.

The installation of inductive charging technology for public, industrial, and home setups must adhere to specific safety standards to protect humans and the environment. By safety regulations and standards, the frequency of IPT varies from 10 kHz to 95 kHz^27^. In this article, we select 86 kHz for design, in compliance with the SAE J2954 standard (81–90 kHz) for EV wireless charging. The circuit in Fig. 1 is simulated, and the results are given in Fig. 3(a)-(d). The DC input voltage is 48 V, and the inverter delivers 48 V and 6.6 A. The transmitter side coil voltage is 47.12 V, and the receiver side voltage is 46.5 V, resulting in a high voltage transfer ratio of 0.98. The currents at the transmitter and receiver sides are 6.4 and 6.2 A, respectively, giving a 0.96 gain. The values can be visualized in Fig. 3(b) and Fig. 3(c). The expected power transfer is 288 W for charging a battery of 48 V,6 A. The output power is 273.58 W, giving DC to DC efficiency of 93.8%, and the coil AC PTE is 94%. To illustrate the performance of the optimized coil system, the voltage gain vs. frequency operation for using an unoptimized coil design is given in Fig. 4.

In IPT applications, the AC to AC PTE with less voltage loss between transmitter and receiver is rarely addressed in the literature, and the main aim of this research is to improve IPT system efficiency by optimizing the coil design by tuning magnetic and geometric parameters. Compared to LEV design utilizing two receiver coils, the voltage and current ratios are relatively low^28^. The proposed optimized compact coil design in the article significantly improves voltage and current gains.

### Overview of the proposed optimization approach

A two-winding circular coil construction holds greater promise for IPT charging, featuring a thin ferrite layer and an iron plate for shielding, as discussed in references^29–31^. The main objective of an effective wireless charging infrastructure is to transfer power effectively across the air gap, and an effective magnetic coil structure is essential for this. Based on our application, the coil size must be compact for lightweight EVs. To find an optimal configuration, the diameter of the coil has to be minimized without compromising the efficiency of the system while maintaining efficient self-inductance L and mutual inductance M between the two coils. The analytical expressions of L and M are given from Eqs. (20)-(23). To estimate and validate, Finite Element Analysis (FEA) analysis with parametric sweep in Maxwell is carried out. The circular loop is defined by the equation given by Neumann’s formula, and the extension of its equation is modified for two coaxial aligned coils with different radii.

20 $$\:L=\frac{{N}^{2}{a}^{2}}{\left(8a+11c\right)}$$

Where, $\:=\frac{{D}_{out}-{D}_{in}}{2}$, $\:c=\frac{{D}_{out}+{D}_{in}}{4}$, $\:{D}_{out}={D}_{in}+2w+\left(sp+w\right)\left(2w-1\right)$, N- Number of turns of the coil, $\:{{D}_{in},D}_{out\:}$– inner and outer diameter of the coil, $\:sp$ – Spacing between the coils, $\:w$- width of the coil wire.

21 $$\:L={N}^{2}R\:{\mu\:}_{0}{\mu\:}_{r}\left[ln\left(\frac{8R}{a}\right)-2\right]\:$$

Where, a –wire radius, R- loop radius

22 $$\:M=\frac{\mu\:}{4\pi\:}\underset{0}{\overset{2\pi\:}{\iint\:}}\frac{abcos(\varnothing\:-{\varnothing\:}^{{\prime\:}})}{{a}^{2}+{b}^{2}+{d}^{2}-2acos(\varnothing\:-\varnothing\:{\prime\:})}\:d\varnothing\:d{\varnothing\:}^{{\prime\:}}$$

The above equation is simplified, and the expression for the mutual inductance is. $\:M$ two coils with differing radii *a* and *b*, number of turns $\:{N}_{1}$ and$\:\:{N}_{2}$, and distance *d*, given in Eq. (21), this analysis is done in correlation with this theoretical validation and simulation validation.

23 $$\:M=\frac{\mu\:\pi\:{n}_{1}{n}_{2}{a}^{2}{b}^{2}}{\sqrt{\left({a}^{2}+{b}^{2}\right)+{d}^{2}}[{\left(a-b\right)}^{2}+{d}^{2}]}$$

The proposed algorithm, named Taylor-based Firefly and Dove Swarm Optimization (F-DSO) algorithm, is a hybrid optimization method that integrates the Taylor Series expansion with the strengths of the FA and DSO algorithms to balance exploration and exploitation, ensuring effective convergence and avoiding local optima. The F-DSO algorithm leverages the computational efficiency and robustness of DSO while integrating the Firefly algorithm’s ability to explore global search spaces effectively. The algorithm incorporates Taylor series expansion for dynamic adjustments based on real-time electrical design parameters.

The proposed approach is given as the steps below:

Step 1: Specify the key system parameters required for optimization.

- Input voltage: V = 48 V.
- Current: I = 6 A.
- Frequency range: f = 81 kHz to 90 kHz.

Additionally, define the initial geometric constraints:

- Outer diameter constraint: D_out_ ≤ 100 mm.
- Spacing between turns: SP ≥ 0.
- Wire width and turn count: W > 0, *N* > 0.

Step 2: Set the initial values for the design variables.

- Number of turns: N.
- Outer diameter: D_in_.
- Wire width: w.
- Spacing between turns: d.

Step 3: Define the objective function to maximize the quality factor (Q) and efficiency (η).

$$\:{F}_{obj}={w}_{1}Q+{w}_{2}\eta\:$$

Where,

$\:Q$ - quality factor is calculated as: $\:Q$ = $\:\frac{\omega\:L}{R}$

L is the inductance.

R is the equivalent resistance, and $\:\omega\:=2\pi\:f$

η is the efficiency, calculated as: $\:\eta\:=\frac{{P}_{out}}{{P}_{in}}$

$\:{P}_{out\:}$– Power delivered to the load

$$\:{P}_{in}$$ - input power

Step 4: Constraints for optimization D_out_ ≤ 100 mm, d ≥ 0, w > 0, *N* > 0.

Step 5: Perform frequency sweep (simulate the system over the frequency range (f ∈ [81, 90] kHz) to calculate:

- Inductance: L.
- Quality factor: Q.
- Efficiency: η.

Store the results in a dataset for further analysis.

Step 6: Apply optimization algorithm to refine the design variables (N, Din, w, d):

- Generate an initial population of solutions.
- Evaluate the objective function for each solution.
- Update the design variables iteratively based on the algorithm rules.

Step 7: Check if the updated design variables satisfy the constraints:

- Geometric constraints (D_out_ ≤ 100 mm).
- Frequency and power constraints (f ∈ [81, 90] kHz, P_out_ ≥ P_min_)

If any constraints are violated, return to Step 6 for further refinement.

Step 8: Determine whether the algorithm has converged by evaluating the change in the objective function ($\:{F}_{obj}$)

- If the change is below a predefined threshold (e.g., < 0.1%), proceed to the next step.
- If not, return to Step 6 and continue iterations.

Step 9: Once convergence is achieved, display the optimized design parameters:

- Optimal values of N, D_out_, w, d.
- Maximum Q and efficiency (η).

Validate the optimized design through field simulation or experimental measurements to ensure compliance with the system requirements.

### Optimization results and discussion

**Fig. 5:**
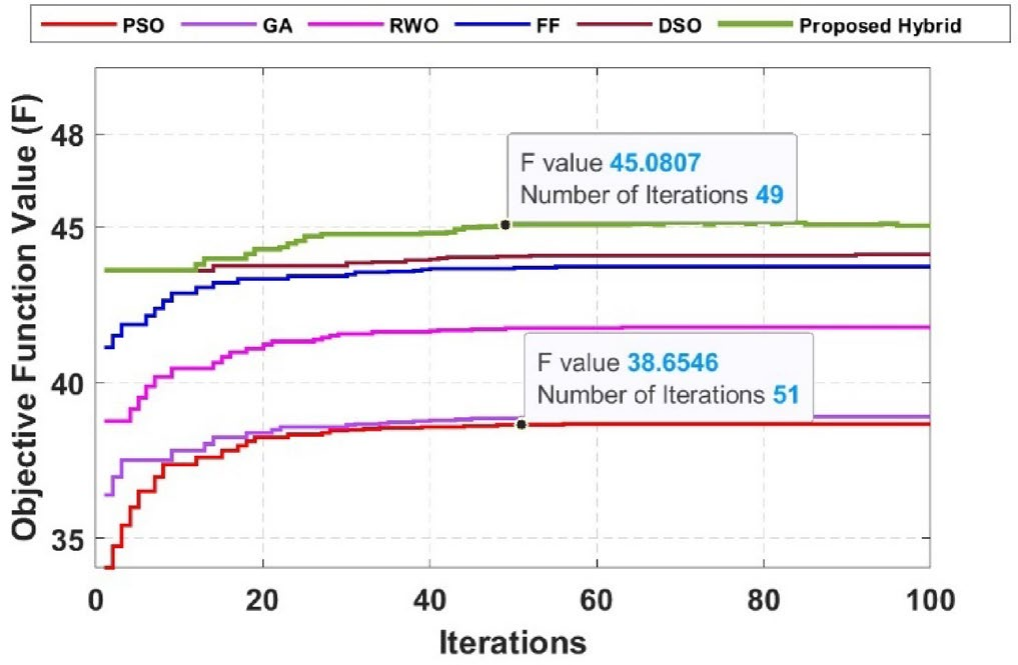
Performance of fitness function ( $\:{F}_{obj}={w}_{1}Q+{w}_{2}\eta\:$ ) coil design optimization process.

The performance metrics of the optimized coil design are analyzed, and the optimization algorithms’ effectiveness is validated, as seen from the convergence plot given in Fig. 5. The hybrid algorithm achieves the highest F value of approximately 45.08 after 49 iterations, demonstrating its superior balance between exploration and exploitation for rapid and robust convergence. DSO performs comparably, attaining an F value of around 44.5. PSO shows a fast initial increase but plateaus early at a lower F, reflecting its limited global search capability. Similarly, GA and RWO converge more slowly, with final F values below 40. FF shows strong early improvement but gradually converges to an F value near 45. The hybrid algorithm’s rapid and accurate convergence underscores its efficiency in optimizing IPT parameters like N, W, and SP (Table 2).

**Table 2:** Optimized parameters for different algorithms.

Algorithm	Efficiency (%)	Quality factor (Q)	Turns (*N*)	Spacing (SP) (mm)	Inner diameter (D_in_) (mm)	Outer diameter (D_out_) (mm)
GA	76	7.20	19	2.8	5.0	143
PSO	79	7.50	16	8.0	6.0	160
RWO	82	7.90	15	2.8	7.0	120
FF	85	8.28	14	2.9	8.0	155
DSO	90	9.0	13	8.9	9.0	160
Hybrid	94	9.78	12	5.6	10.0	110

The optimal parameters for each algorithm are summarized in Table 1, and it shows that the hybrid algorithm achieves the highest. $\:Q$ and *η* values while requiring the fewest iterations, affirming its superior optimization capability.

## Hardware prototype results for optimized coil design

**Fig. 6:**
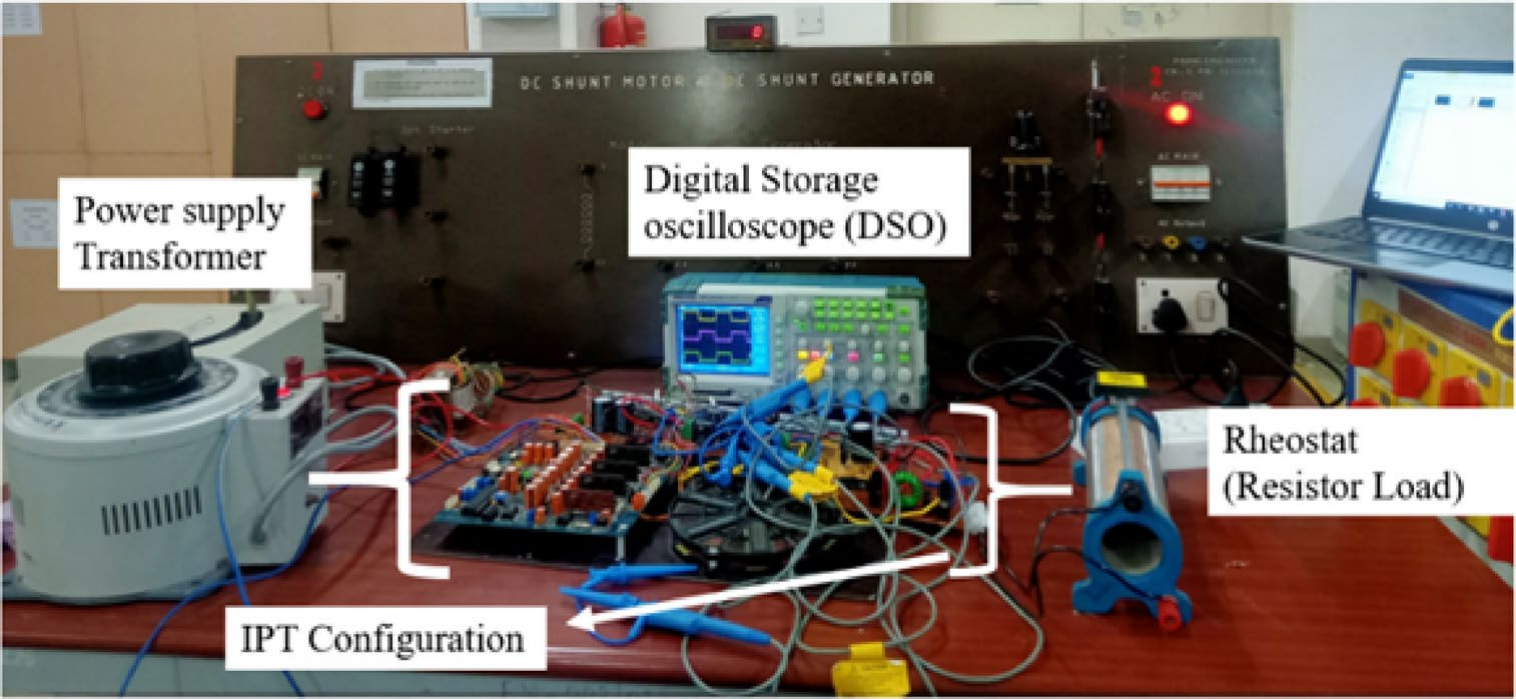
Experiment setup.

The hardware setup is developed based on the configuration depicted in Fig. 1. The power rating depends on the availability of power sources and component cost. Therefore, a prototype is designed with 48 V, and an air gap range of 4–10 mm^32^. The prototype, as shown in Fig. 6, is constructed in a compact form to minimize losses, and the coils are positioned with a 10 mm air gap.

**Fig. 7:**
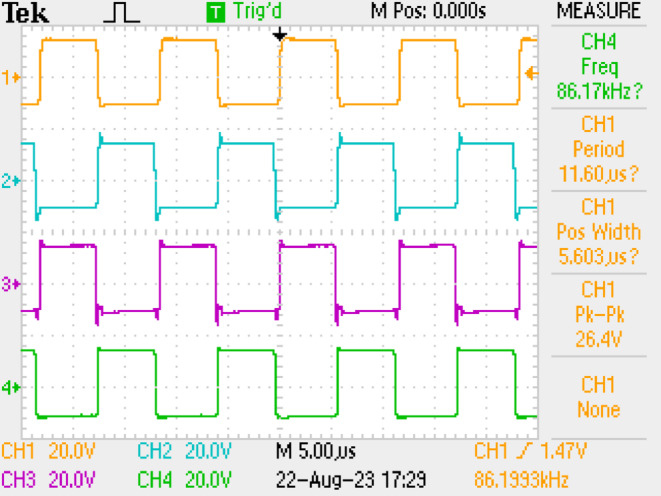
Device voltages (V_ds_) of the inverter.

**Fig. 8:**
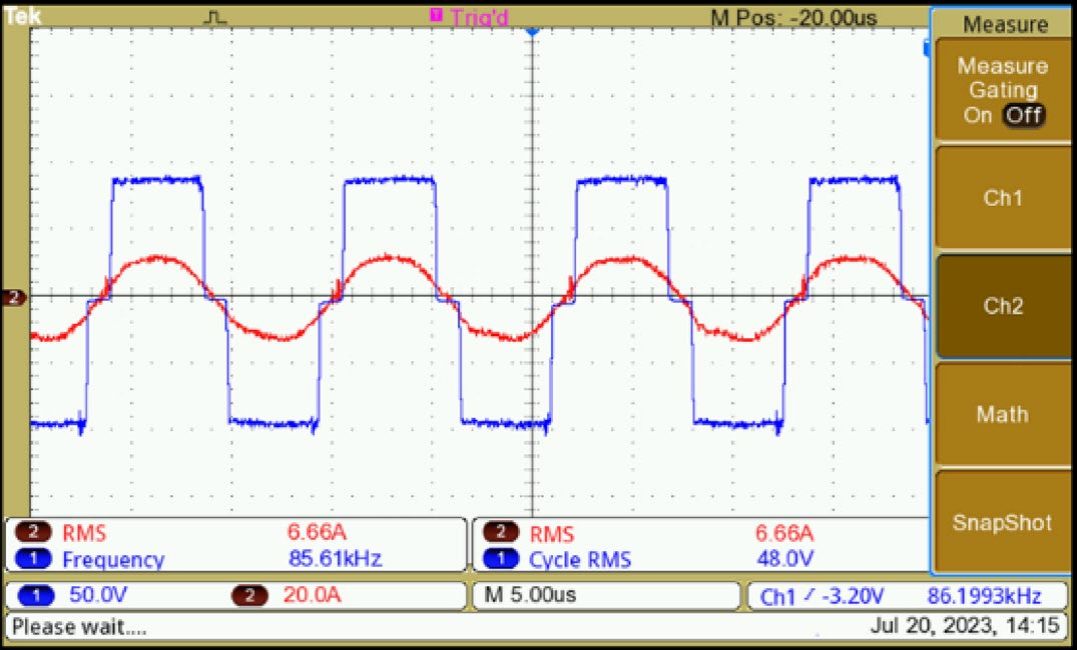
Primary side HF inverter voltage and current.

**Fig. 9:**
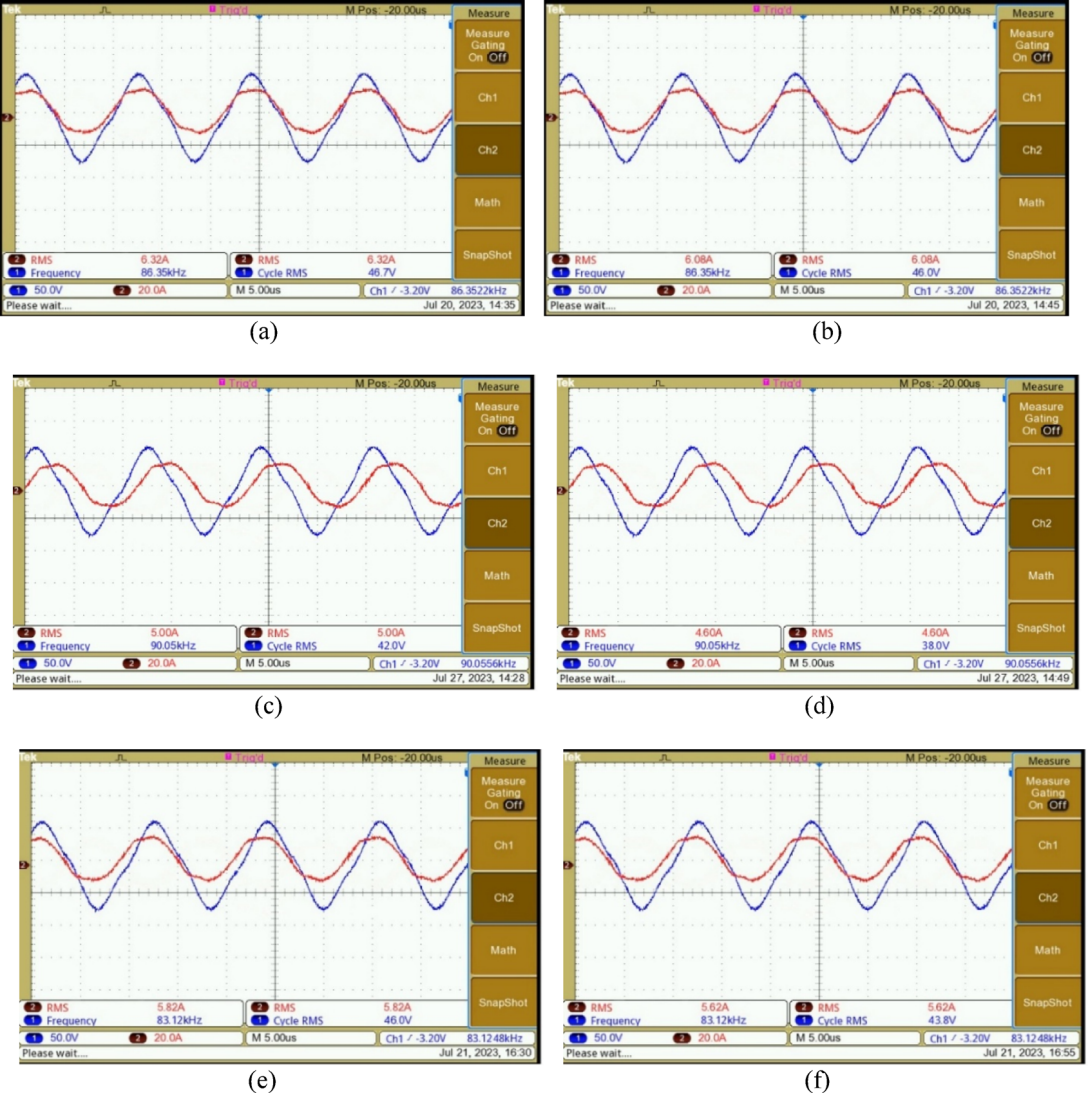
(**a**) Primary side voltage at operating resonant frequency (86 kHz) (**b**) secondary side voltage at operating resonant frequency (86 kHz) (**c**) primary side voltage above resonant frequency (90 kHz) (**d**) secondary side voltage above resonant frequency (90 kHz) (**e**) primary side voltage below resonant frequency (83 kHz) (**f**) secondary side voltage below resonant frequency (83 kHz).

The primary source for energizing the coils is the HF inverter. This inverter is integrated with control and driver circuits to facilitate precise pulse triggering. The driver IC IR2110 is used with 20N60D MOSFET, while the Texas Instruments TMS320F28379D controller interfaces seamlessly with MATLAB Simulink’s pulse generation block. The switching performance is monitored using a Digital Storage Oscilloscope (DSO). The inverter switches need to be activated with a deliberate delay to prevent a short circuit within the same leg of the bridge structure. To achieve this, a delay of 43% is introduced by analyzing the switch transitions. The switch voltages of the HF inverter (V_ds1_ to V_ds4_) are shown in Fig. 7. Figure 8 is the voltage and current response of the inverter. These waveforms are recorded before the application of the compensation circuit. The response of the system, with the inclusion of the compensation LC circuit, is illustrated in Fig. 9(a)-(f). The current lags the voltage below the resonant frequencies, and the current leads the voltage above the resonant frequencies. The voltage and current values are given in Table 3 for various frequencies. Notably, the DC voltage transfer exhibits high efficiency as seen in the output with 45.6 V at 5.6 A (253 W) with an input voltage of 48 V at 6.6 A (288 W), achieving minimal power loss with PTE of 94%.

**Table 3:** Experimental values: current and voltage variable frequency operation.

Variable parameter	Values			
Frequency	Primary voltage	Secondary voltage	Primary current	Secondary current
81 kHz	45.4 V	42.4 V	5.32 A	4.82 A
83 kHz	46 V	43.8 V	5.82 A	5.62 A
86 kHz	46.7 V	46 V	6.32 A	6.08 A
87 kHz	45.5 V	43.6 V	6.02 A	5.8 A
89 kHz	43.4 V	40 V	5.5 A	5 A
90 kHz	42 V	5 A	38 V	4.6 A

## Conclusion

A detailed analysis of an optimized coil configuration for low-power and compact wireless power transfer (WPT) applications is presented in this study. The primary focus was to minimize coil diameter while maintaining efficient self-inductance and mutual inductance by optimal tuning of coil geometry parameters. The inductive power transfer (IPT) system operates at 86 kHz, also its design performance is validated through variable frequency variation. An extensive review of existing literature, design equations, and simulation models guided to development of a prototype charging infrastructure using an LC-compensated optimized coil design for a 48-volt battery system. The Taylor-Dove swarm hybrid optimization algorithm is implemented in this paper, which iteratively adjusts the coil diameter and other parameters to identify a compact and efficient configuration. The experimental results correlate with the theoretical predictions and simulation models, with the hardware achieving a PTE of 94%, a voltage gain of 0.93, and a current gain of 0.96 across a 10 mm air gap. The compact IPT charging infrastructure in this paper can be used in LEVs and other low-power scenarios. The successful implementation and testing of the prototype highlight the potential of the optimized coil design to enhance the efficiency and compactness of wireless charging systems. It provides a robust foundation for future research in compact, high-efficiency wireless charging solutions. Future research will focus on further enhancing the coil design, exploring higher power levels, and optimizing other components of the IPT system to achieve even greater efficiency and performance.

## Data Availability

All data generated or analyzed during this study are included in this published article. Additional information is available from the corresponding author upon reasonable request.
